# A new species of the genus *Arrup* from a limestone cave in Akiyoshi-dai, Western Japan (Chilopoda, Geophilomorpha, Mecistocephalidae)

**DOI:** 10.3897/zookeys.830.33060

**Published:** 2019-03-14

**Authors:** Sho Tsukamoto, Satoshi Shimano, Takashi Murakami, Shimpei F. Hiruta, Takeshi Yamasaki, Katsuyuki Eguchi

**Affiliations:** 1 Systematic Zoology Laboratory, Graduate School of Science, Tokyo Metropolitan University, Minami-osawa 1-1 Hachioji-shi, Tokyo, 192-0397, Japan Tokyo Metropolitan University Tokyo Japan; 2 Science Research Center, Hosei University, Fujimi 2-17-1 Chiyoda-ku, Tokyo, 102-8160, Japan Hosei University Tokyo Japan; 3 Cultural Property Protection Division, Board of Education, Mine City Office, Akiyoshi 5353-1 Shuho-cho, Yamaguchi, 754-0511, Japan Board of Education, Mine City Office Yamaguchi Japan; 4 Center for Molecular Biodiversity Research, National Museum of Nature and Science, Tokyo, Amakubo 4-1-1 Tsukuba-shi, Ibaraki, 305-0005, Japan National Museum of Nature and Science Tokyo Japan

**Keywords:** Arrupinae, Chilopoda, Kagekiyo-ana, limestone, taxonomy, 18S rRNA gene

## Abstract

*Arrupakiyoshiensis* Tsukamoto & Shimano, **sp. n.** is described from a limestone cave, Kagekiyo-ana, in Akiyoshi-dai, one of the largest karst regions in Japan, Yamaguchi prefecture. It is distinguishable from 14 valid named congeners by some unique characteristics including entire areolation on the cephalic pleurite, elongation of distal part of female gonopod, and a tubercle on forcipular segment II. In addition, the 18S rRNA gene sequences of *A.akiyoshiensis* Tsukamoto & Shimano, **sp. n.** and *A.ishiianus*, one of the most morphologically similar species, differed by four bp out of 1821 bp. The fact that only troglobionts and troglophilic species are found in the collection site suggests that this new species might be a cave-dweller.

## Introduction

Centipedes are for the most part soil-dwellers, and common in various habitats such as forests, grasslands, coastal areas and so on (Lewis 1981). Although the most of soil-dwelling animal taxa have troglobionts species, few troglobiotic centipedes have so far been recorded (Chagas-Jr and Bichuette 2018). Especially, despite high adaptation for subterranean habitats, only two troglobionts species are hitherto known in Geophilomorpha: *Geophiluspersephones* Foddai & Minelli, 1999 and *Geophilushadesi* Stoev, Akkari, Komerički, Edgecombe & Bonato, 2015. Both of them have unusual traits, which are common among troglobiotic arthropods (exceptionally elongated antennae, legs, and claws) (Foddai and Minelli 1999, Stoev et al. 2015). In Japan, several centipede species can be found in both the inside and outside cave, and Shinohara (1966) referred two species were considered to be troglobiotic centipedes; *Brachygeophiluspolyporus* Takakuwa, 1942 (Geophilomorpha) and *Monotarsobiusminor* Takakuwa, 1942 (Lithobiomorpha). Commonly, the troglobiotic fauna has a high proportion of endemic species in each cave or cave group (Gibert and Deharveng 2002; Christman et al. 2005). Many endemic species with small geographic ranges may occur in isolated caves (Barr Jr and Holsinger 1985); therefore, the inventory of the troglobiotic fauna is important to clarify the formulation of endemism.

Akiyoshi-dai, where is a one of the largest karst regions in Japan, has a spread of 16 km in northeast direction and 6 km northwest direction, with more than 400 limestone caves (Fujii 2009). Thirteen invertebrate species are endemic to the area (Kuramoto 1980). For Chilopoda, 13 species are recorded from Akiyoshi-dai, but all of them are not endemic species (Kuramoto 1980). In the course of our recent survey of cave invertebrates in Kagekiyo-ana cave of Akiyoshi-dai, six individuals belonging to the genus *Arrup* Chamberlin, 1912 were collected, and later confirmed as a new species based on our careful morphological examination and comparison with 14 valid named congeners (Uliana et al. 2007, Bonato et al. 2016) by using cephalic capsule, mandible, maxillae, the number of coxal pore and genital segments. We herein describe this species as *A.akiyoshiensis* sp. n.

## Materials and methods

**Figure 1.**
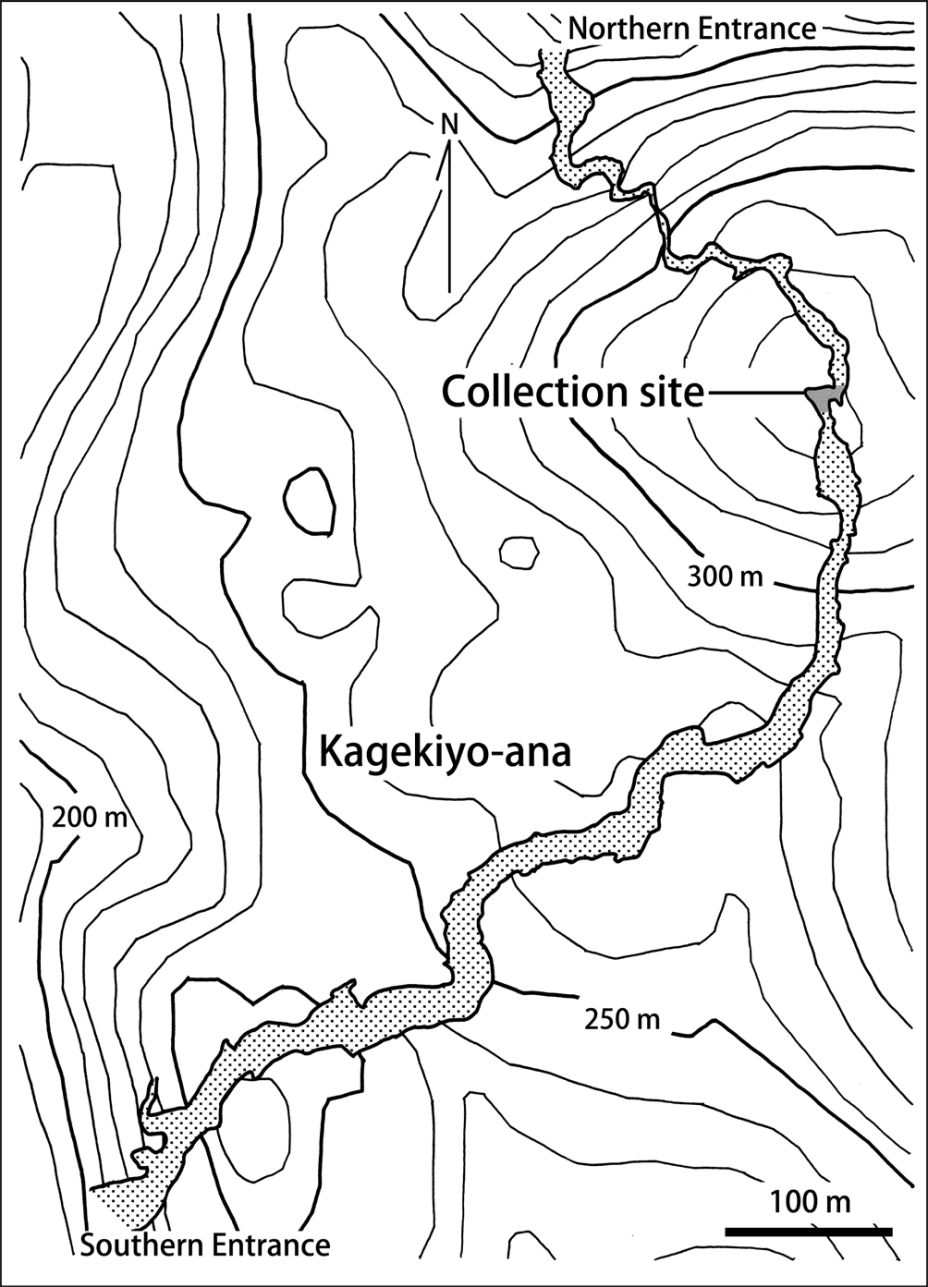
A map of Kagekiyo-ana cave. Contour lines are shown with altitudes every 50 m.

Two adult female specimens and four juvenile specimens of *A.akiyoshiensis* sp. n. were collected by hand under rocks inside Kagekiyo-ana cave (a limestone cave; 34°17.50'N, 131°20.00'E), in Akiyoshi-dai (a karst region), Mitou-cho, Mine-shi, Yamaguchi Prefecture, Japan. The exact position of the collection site is shown in Fig. 1. This was 130 m below the surface, 500 m from the northern entrance, and 900 m from the southern entrance of the cave. In addition, one specimen of *A.ishiianus* Uliana, Bonato & Minelli, 2007 from Imperial Palace, Tokyo was used for comparing morphology and 18S rRNA gene sequence with *A.akiyoshiensis* sp. n. Each specimen examined in the present study is specified by its specimen identification number, in the form “TS-YYYYMMDD-XX”; where TS is an abbreviation of the first author, Tsukamoto Sho; YYYYMMDD designates the date on which the specimen was collected; and XX is the identification number given to each specimen collected on that date (e.g., TS-20180330-01). All specimens are deposited at the Collection of Myriapoda, National Museum of Nature and Science, Tokyo (**NMST**).

Specimens were observed and drawn in lactic acid on temporary cavity slides using a Nikon Eclipse E600 microscope, and were then mounted with Hoyer’s medium (gum arabic, chloral hydrate and glycerol). Some characters were photographed by using Panasonic LUMIX DMC-GX8 and Canon EOS Kiss X9, and focus stack images were produced from a series of pictures at different focal planes by Helicon Focus Pro version 6.6.1 on a desktop PC. Note that the external shape might be slightly distorted when immersed in lactic acid because of expansion of internal tissue. Besides, specimens were measured with their each part mounted with Hoyer’s medium in order to avoid distortion of the external shape. The morphological terminology used below is mainly based on Bonato et al. (2010).

Genomic DNA was extracted from part of the appendage using a DNeasy Blood & Tissue Kit (Qiagen), with modifications from Johnson et al. (2004). An appendage of each specimen was incubated at 55 °C for 48 h to lyse the tissue. Before each lysis mixture was pipetted into a spin column, the exoskeleton was retrieved and preserved in 100% Ethanol.

Table 1 lists all primers used in this study. Partial sequences of 18S rRNA gene were amplified by polymerase chain reactions (PCR) using the primer sets, 18S-F1 and 18S-R9 (Yamaguchi and Endo 2003). The PCR amplification was performed in a Thermal Cycler Dice (Takara) in a 10 μl volume containing 0.5 μl of template solution, 2 mM MgCl_2_, 2.5 mM each dNTP, 10 pmol each primer, and 0.25 U Ex Taq polymerase Hot Start version (Takara) in 1× buffer provided by the manufacturer. Amplification conditions were 95 °C for 2 min; 35 cycles of 95 °C for 30 sec, 50 °C for 30 sec, and 72 °C for 2 min; and 72 °C for 7 min.

**Table 1.** Primers used in this study.

Gene	Name	Sequence (5'–3')	Direction	Source	Used for PCR
18S rRNA	18S-F1	TACCTGGTTGATCCTGCCAG	forward	Yamaguchi and Endo (2003)	*
	18S-F2	CCTGAGAAACGGCTRCCACAT	forward	Yamaguchi and Endo (2003)	
	18S-F3	GYGRTCAGATACCRCCSTAGTT	forward	Yamaguchi and Endo (2003)	
	18S-F4	GGTCTGTGATGCCCTYAGATGT	forward	Yamaguchi and Endo (2003)	
	18S-R6	TYTCTCRKGCTBCCTCTCC	reverse	Yamaguchi and Endo (2003)	
	18S-R7	GYYARAACTAGGGCGGTATCTG	reverse	Yamaguchi and Endo (2003)	
	18S-R8	ACATCTRAGGGCATCACAGACC	reverse	Yamaguchi and Endo (2003)	
	18S-R9	GATCCTTCCGCAGGTTCACCTAC	reverse	Yamaguchi and Endo (2003)	*

Amplification products were purified with the ExoSAP-IT kit (Thermo Fisher Scientific). All nucleotide sequences were determined by direct sequencing using a BigDye Terminator Cycle Sequencing Kit ver. 3.1 with an ABI 3500XL automated sequencer (Thermo Fisher Scientific). The amplification primers and internal primers were used in sequencing 18S rRNA gene. Nucleotide sequences were assembled and edited with MEGA7 (Kumar et al. 2016). Sequences have been deposited in DDBJ/EMBL/GenBank database under accession numbers LC460298–LC460301 (Table 2).

**Table 2.** GenBank accession numbers of *Arrup* sequence data.

Species	Collection site	Specimen identification no.	Accession no.
* Arrup akiyoshiensis *	Kagekiyo-ana, Yamaguchi	TS-20180330-01 (holotype)	LC460298
* Arrup akiyoshiensis *	Kagekiyo-ana, Yamaguchi	TS-20180418-01 (paratype)	LC460299
* Arrup akiyoshiensis *	Kagekiyo-ana, Yamaguchi	TS-20180418-02	LC460300
* Arrup ishiianus *	Imperial Palace, Tokyo	TS-20090729-01	LC460301

## Taxonomy

### Family Mecistocephalidae Bollmann, 1893

#### Genus *Arrup* Chamberlin, 1912

##### Arrup akiyoshiensis

Taxon classificationAnimaliaGeophilomorphaMecistocephalidae

Tsukamoto & Shimano sp. n.

http://zoobank.org/6B8C8441-CD7C-4F1C-9280-1DF1D5EDB994

Figs 2 , 3 , 4 , 5 , 6 , 7 , 8 , 9 , Tables 3 , 4 , 5 , 6

###### Type Material.

**Holotype** 1 female, Kagekiyo-ana, Mitou Town (Mitou-cho), Mine City (Mine-shi), Yamaguchi Prefecture, Japan, 30^th^ of March 2018, coll. Takashi Murakami (labeled as TS-20180330-01). **Paratype** 1 female, Kagekiyo-ana, Mitou Town (Mitou-cho), Mine City (Mine-shi), Yamaguchi Prefecture, Japan, 18^th^ of April 2018, coll. Takashi Murakami (labeled as TS-20180418-01).

###### Etymology.

The species name is derived from the name of Akiyoshi-dai Karst region, which includes the type locality.

###### Diagnosis.

*Arrupakiyoshiensis* sp. n. can be distinguished from the all named congeners by a combination of the following morphological characteristics: frontal line curved; seven pectinate lamellae in mandible; comma-shaped distal lobe of coxal projection in first maxillae; a tiny tubercle on outer-distal corner of each article of the telopodite; distal article of the telopodite of the second maxillae without claw; the well-developed tooth of forcipular article I; the triangular basal tooth in tarsungulum; the poison calyx overreaching forcipular article I; 31–35 pores on lateral and ventral sides on coxopleura.

###### Description.

Measurements of the holotype (adult female, TS-20180330-01) are followed by those of 1 paratype (adult female, TS-20180418-01) in parentheses. Body length 36.0 (34.5) mm, maximum body width 1.0 (0.95) mm, cephalic plate length 1.45 (1.30) mm, maximum cephalic plate width 0.92 (0.78) mm.

**Figure 2.**
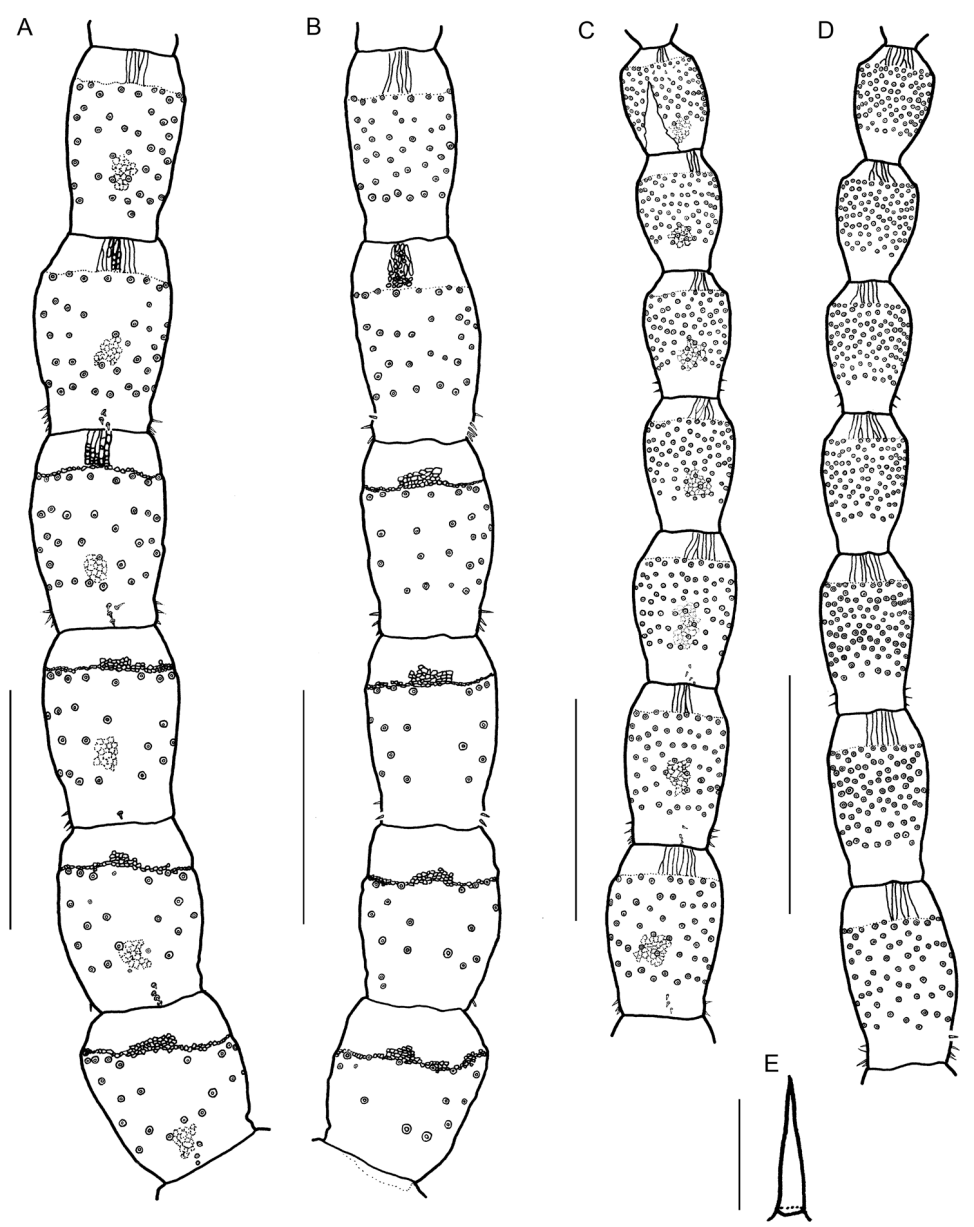
*Arrupakiyoshiensis* sp. n., holotype (TS-20180330-01), **A** right antennal article, I–VI, dorsal **B** right antennal article, I–VI, ventral **C** right antennal article, VII–XIII, dorsal **D** right antennal article, VII–XIII, ventral **E** a pointed sensillum on the dorsal side of article XIII. Setae are not drawn, only their sockets. Scale bars: 500 µm (**A–D**); 10 µm (**E**).

**Figure 3.**
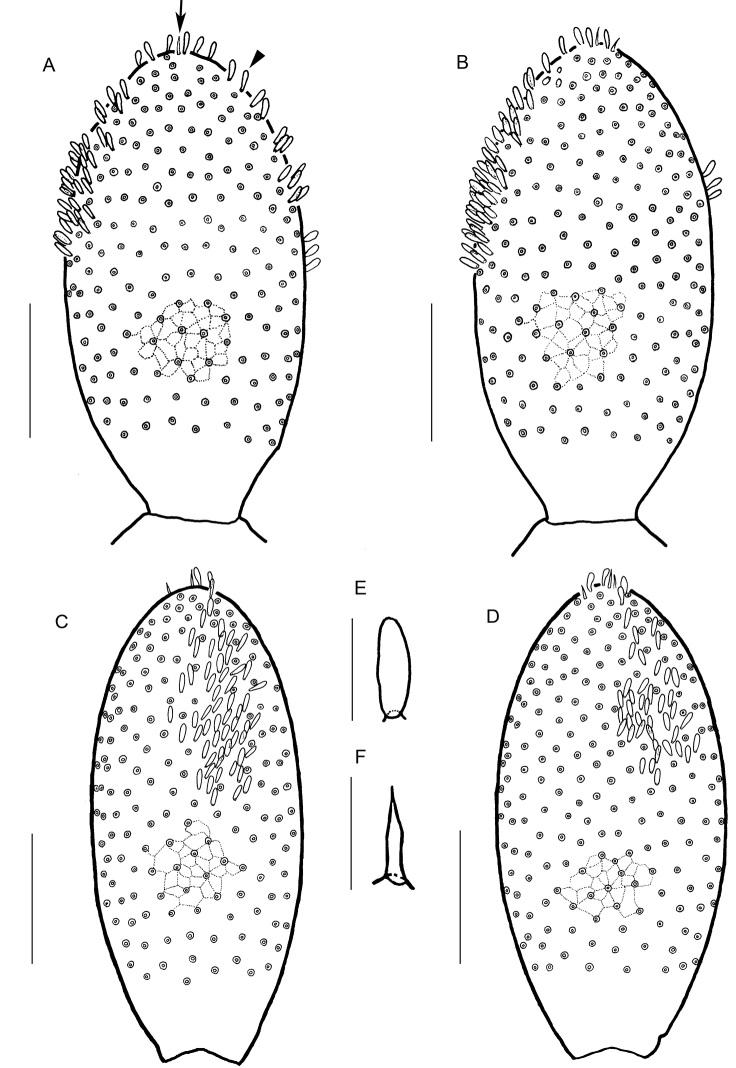
*Arrupakiyoshiensis* sp. n., holotype (TS-20180330-01), **A** right antennal article, XIV, dorsal **B** right antennal article, XIV, ventral **C** right antennal article, XIV, outer-lateral **D** right antennal article, XIV, inner-lateral **E** a claviform sensillum on the antennal article XIV (triangle in fig. 9) **F** a pointed sensillum on the antennal article XIV (arrow in fig. 9). Setae are indicated only with sockets. Scale bars: 100 µm (**A–D**); 25 µm (**E**); 12.5 µm (**F**).

Antenna (Figs 2A–E, 3A–F, 7D, Tables 3–5) length 3.4 times as long as cephalic plate length. All articles weak areolate, except anterior margin; anterior margin of articles I to IV well areolate. Articles I to V slightly asymmetrical, with internal margin longer than external margin. Articles VI to XIV symmetrical. Setae on articles I to XIV spiniform, arranged uniformly (Figs 2A–D, 3A–D, Table 3). Distodorsal and distoventral surfaces of articles II, V, IX, and XIII with 1–7 small pointed sensilla (Fig. 2E, Table 5). Article XIV with 96–101 claviform sensilla on outer-lateral and inner-lateral sides (Figs 3A–E, triangle in Fig. 3A, Table 4), with 6–9 pointed sensilla on the tip (Figs 3A–D, F, arrow in Fig. 3A, Table 5).

**Table 3.** Number of right antennal setae of *Arrupakiyoshiensis* sp. n., holotype (TS-20180330-01).

**Antennal article**	**I**	**II**	**III**	**IV**	**V**	**VI**	**VII**	**VIII**	**IX**	**X**	**XI**	**XII**	**XIII**	**XIV**
Number of Setae	45	53	57	73	98	102	140	155	207	201	215	207	194	343

**Table 4.** Number of claviform sensilla on antennal article XIV of *Arrupakiyoshiensis* sp. n., holotype (TS-20180330-01), paratype (TS-20180418-01).

**Side of antenna**	**Right**		**Left**	
	**Inner-lateral**	**Outer-lateral**	**Inner-lateral**	**Outer-lateral**
Holotype (TS-20180330-01)	43	58	36	60
Paratype (TS-20180418-01)	38	61	n/a	n/a

Note n/a: Antennal article XIV left was lost.

**Table 5.** Number of antennal pointed sensilla of *Arrupakiyoshiensis* sp. n., holotype (TS-20180330-01).

**Antennal article**	**I**	**II**	**III**	**IV**	**V**	**VI**	**VII**	**VIII**	**IX**	**X**	**XI**	**XII**	**XIII**	**XIV**
Right, dorsal	0	3	0	0	8	0	0	0	4	0	0	0	3	6
Right, ventral	0	2	0	0	5	0	0	0	4	0	0	0	3	
Left, dorsal	0	1	0	0	7	0	0	0	7	0	0	0	3	9
Left, ventral	0	2	0	0	5	0	0	0	5	0	0	0	3	

**Figure 4.**
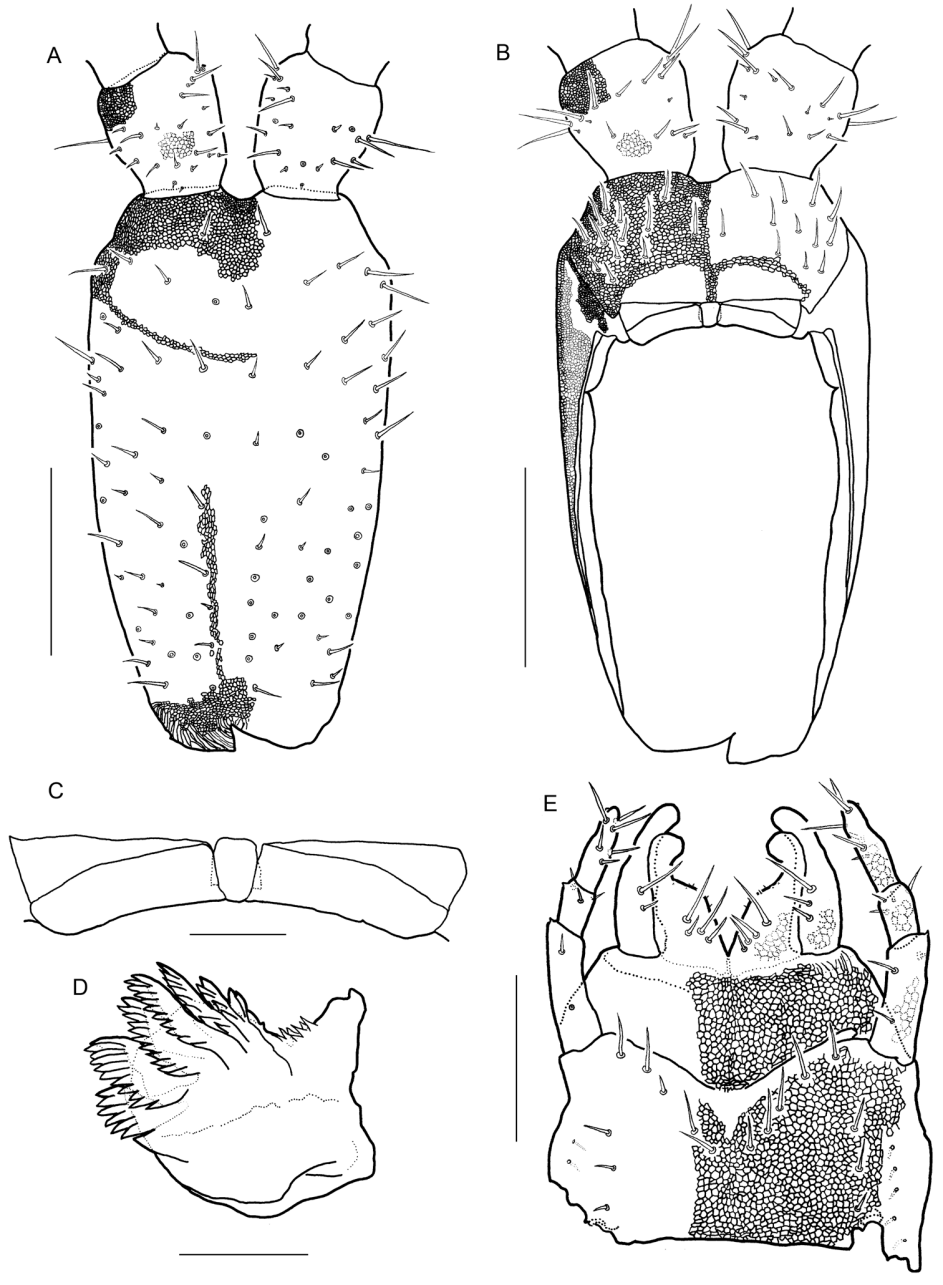
*Arrupakiyoshiensis* sp. n., holotype (TS-20180330-01), **A** cephalic plate, dorsal **B** clypeus and clypeal pleurite, ventral **C** labrum, ventral **D** right mandible, dorsal **E** maxillae complex, ventral. Note that **A–C** are distorted by the effects of lactic acid. Scale bars: 500 µm (**A, B**); 100 µm (**C, D**); 250 µm (**E**).

Cephalic plate (Figs 4A, 8A) 1.5 times as long as wide. Transverse suture present. Paramedian sulci present. Lateral margins almost straight and convergent backwards; anterior margin convex; posterior margin straight. Surface areolated; proximal and distal scutes clearly marked. Setae arranged nearly symmetrically.

Clypeus (Figs 4B, 8B) 1.5 times as wide as long. Clypeal area absent. Paraclypeal sutures complete, strongly convergent backwards. Two clypeal plagulae not contacting with the paraclypeal sutures; remaining clypeal parts uniformly areolate, with setae arranged in two groups.

Labrum (Fig. 4C) consisted of three pieces. Side pieces divided into anterior and posterior alae with convexed chitinous line. Longitudinal stripes on the posterior alae absent. Anterior margin of side pieces almost straight. Internal margins of side pieces convergent backward, but not bordered directly with each other. Posterior margin of side pieces convex. Mid-piece 1.1 times as long as wide.

Cephalic pleurite (Figs 4B, 8C) with areolation entirely except a part of anterior region; scutes clearly marked along anterior margin, lateral margin, and paraclypeal suture. Spicula absent. Setae absent. Stilus well chitinized.

Mandible (Fig. 4D, Table 6) with seven pectinate lamellae. Lamellar teeth sharp; 2–15 teeth present in each lamella (Table 6); anterior tooth gradually longer than posterior one in each lamella.

**Table 6.** Number of the pectinate lamellae of *Arrupakiyoshiensis* sp. n., holoype (TS-20180330-01) and paratype (TS-20180418-01).

Pectinate lamellae	Right							Left						
	I	II	III	IV	V	VI	VII	I	II	III	IV	V	VI	VII
Holotype (TS-20180330-1)	6	11	11	11	8	5	2	6	11	11	11	8	5	2
Paratype (TS-20180418-1)	6	11	15	12	8	4	2	6	11	13	9	3	4	–*

* pectinate lamella VII of paratype was broken.

First maxillae (Figs 4E, 8D) undivided, without mid-longitudinal suture in coxosternite, convergent forward; anterior corners not projecting; ventral surface areolate, except for anterior and lateral margins; setae absent. Coxal projection well developed, with six spines on each internal margin and 4–5 setae at the each middle position. Basal part of medial projection round, with distal lobe; distal lobe clavate as comma-shaped. Basal part 1.7 times as long as distal lobe.

Second maxillae (Figs 4E, 8D) undivided, without mid-longitudinal suture in coxosternite; 5 + 5 setae arranged along the anterior margin, 3 + 3 setae on lateral side. Isthmus areolate. Anterior and posterior margins concave. Lateral margins parallel. Telopodites triarticulated, reaching the telopodite of first maxillae. Claw of the telopodite absent. A tiny tubercle present on outer-distal corner of each article. Article I 2.8 times as long as wide; article II 1.6 times as long as wide; article III 3.2 times as long as wide.

**Figure 5.**
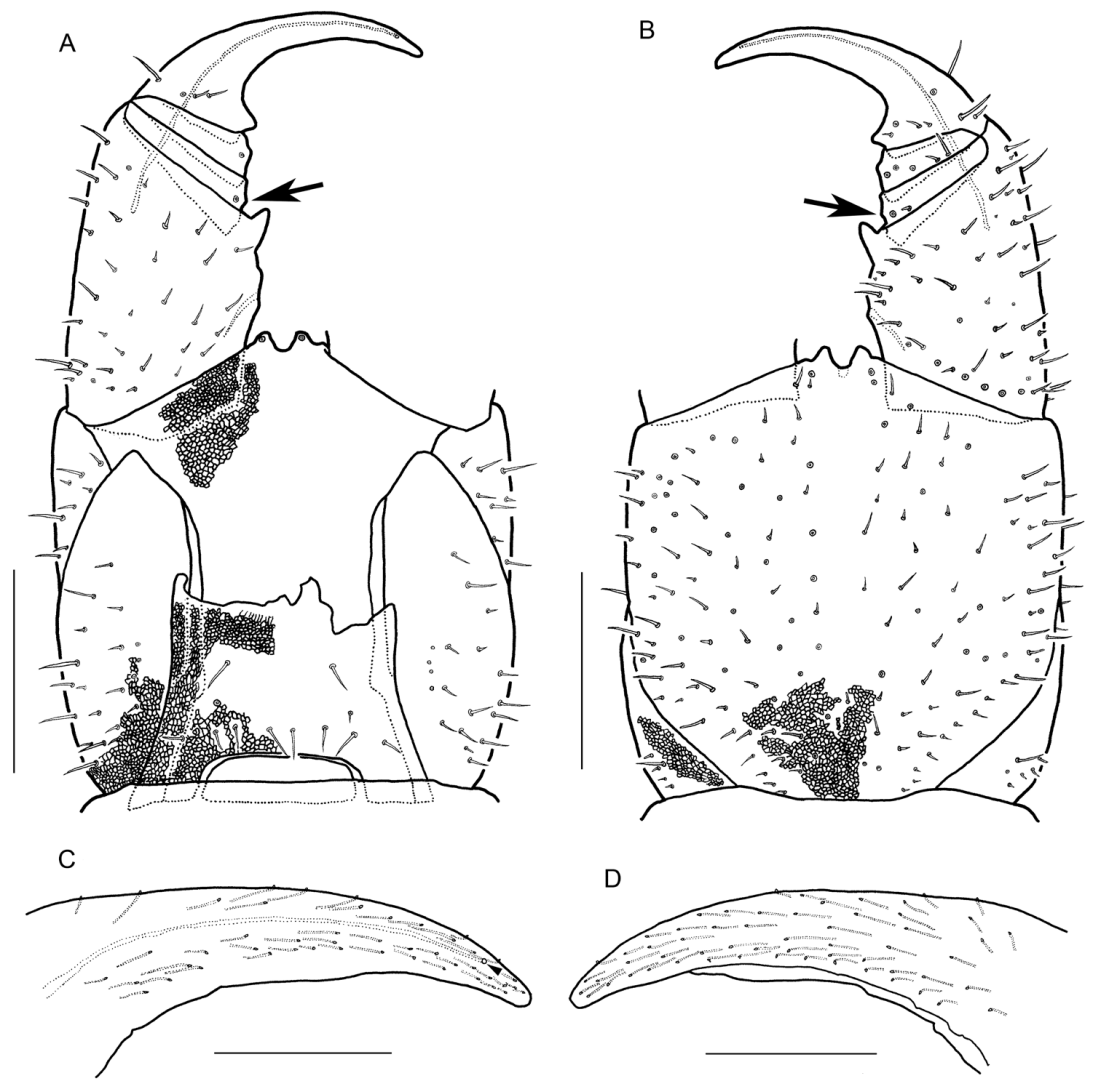
*Arrupakiyoshiensis* sp. n., **A, B** paratype (TS-20180418-01) **C, D** holotype (TS-20180330-01) **A** forcipular segment and left forcipule, dorsal **B** forcipular segment and left forcipule, ventral **C** claw of left tarsungulum, dosral **D** claw of left tarsungulum, ventral. Scale bars: 500 µm (**A, B**); 200 µm (**C, D**).

Forcipular segment (Figs 5A–D, 7B, C, 9A, B) with setae both on dorsal and ventral surface; setae arranged almost symmetrically. Coxosternite with distinct 1 + 1 projections in anterior margin. Chitinous lines absent. Forcipular tergite trapeziform. When telopodites closed, tarsungulum reaching anterior margin of cephalic plate. Article I 1.9 times as long as wide, with a well-developed pointed tooth at the distal internal corner. Article II 0.40 times as long as wide, with a tubercle at the internal margin (arrows in Fig. 5A, B). Article III 0.37 times as long as wide, with a tubercle at the internal margin. Tarsungulum with a triangular basal denticle. Claw of tarsungulum with numerous tiny sensilla. Calyx of poison gland overreaching article I. Duct opening of poison gland on dorsal tip of tarsungulum (triangle in Fig. 5C).

**Figure 6.**
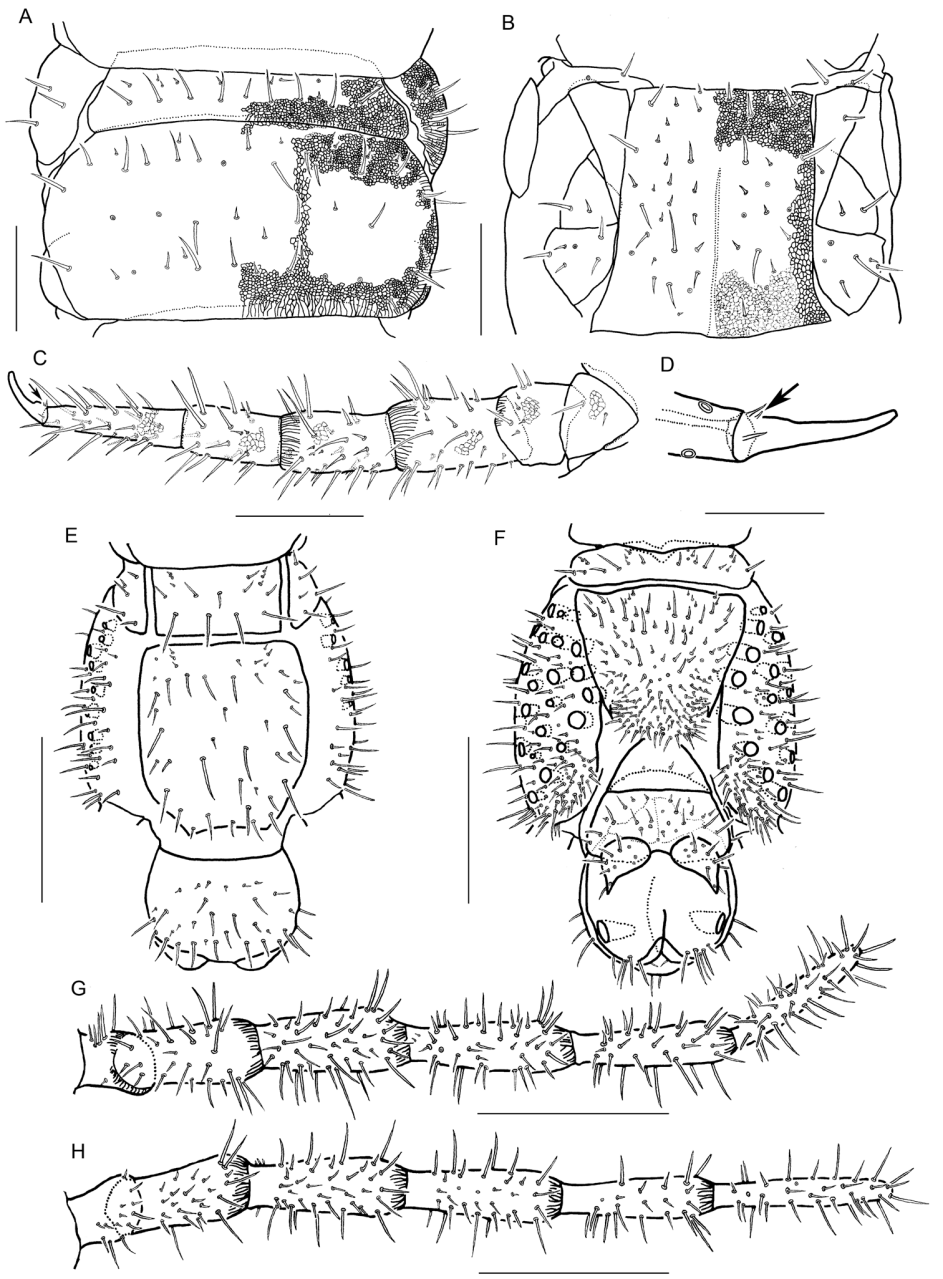
*Arrupakiyoshiensis* sp. n., holotype (TS-20180330-01), **A** four leg-bearing segments, dorsal **B** four leg-bearing segments, ventral **C** left leg (pair 4), ventral **D** claw of right leg (pair 4), lateral **E** last leg-bearing and postpedal segments, dorsal **F** last leg-bearing and postpedal segments, ventral **G** right telopodite of last leg-bearing segment, dorsal **H** right telopodite of last leg-bearing segment, ventral. Scale bars: 300 µm (**A–C**); 100 µm (**D**); 500 µm (**E–H**).

Leg-bearing segments (excepting last leg-bearing segment) (Fig. 6A–D) without pore field on sternites. Median longitudinal sulcus present on sternites I–XVII. Forty-one leg-bearing segments in both the holotype and paratype. All legs weakly areolate. First pair of legs much shorter than the others. All leg claws with anterior and posterior accessory spines; posterior one with a subsidiary spine at its bottom (arrows in Fig. 6C, D).

Last leg-bearing segment (Figs 6E–H, 9C, D) with numerous setae both on tergite and sternite; setae arranged almost symmetrically. Sternite as long as wide, sub-triangular, with posterior margin round. Tergite sub-pentagonal. Coxopleura with 31–35 pores on lateral and ventral sides. Telopodite having six articles, but without claw.

Postpedal segment (Figs 6E, F, 9C, D) with setae on each segment; setae arranged almost symmetrically. Female gonopod uniarticulate; distal part elongate. Anal pore opened laterally.

**Figure 7.**
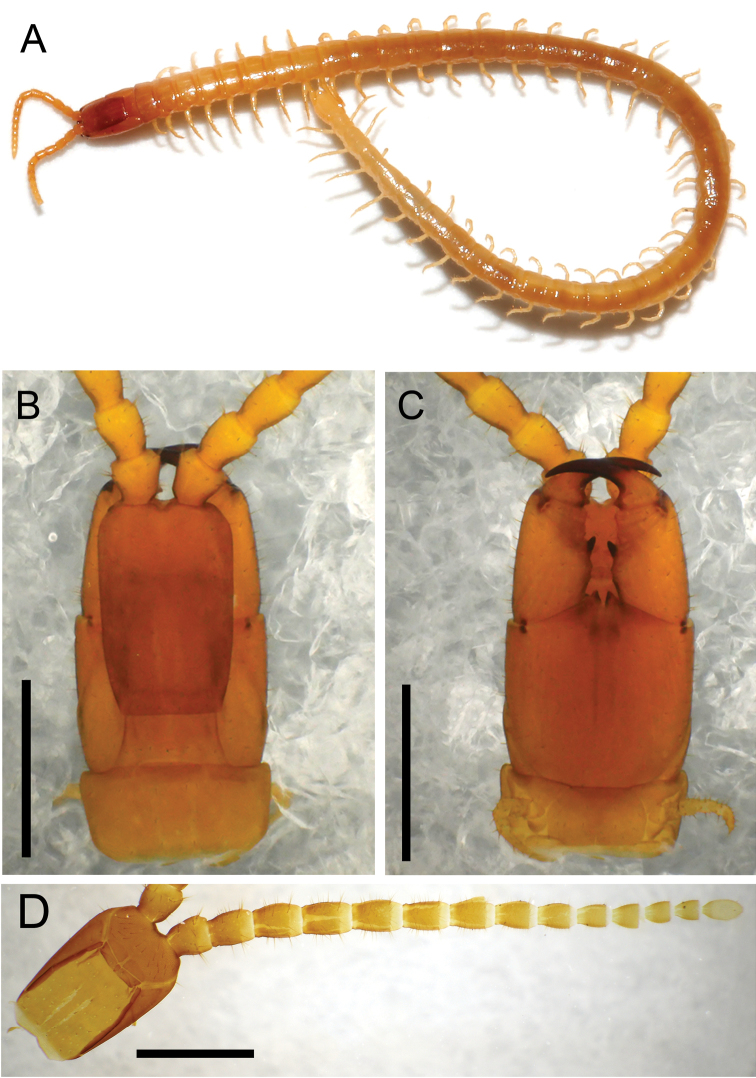
*Arrupakiyoshiensis* sp. n., **A–C** paratype (TS-20180418-01) **D** holotype (TS-20180330-01) **A** whole body, dorsal **B** head and forcipular segment, dorsal **C** head and forcipular segment, ventral **D** head and left antenna, ventral. Scale bar: 1 mm (**B–D**).

Coloration (Fig. 7A). Head and forcipular segment pale ocher; other body segments whitish yellow, without dark patches.

**Figure 8.**
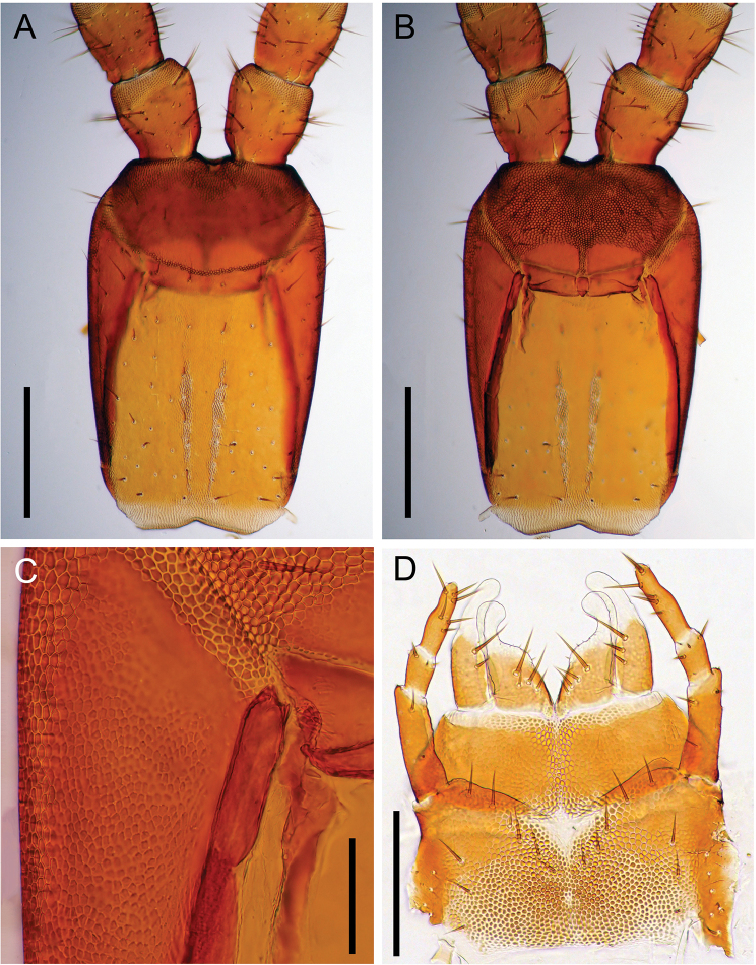
*Arrupakiyoshiensis* sp. n., holotype (TS-20180330-01), **A** cephalic plate, dorsal **B** clypeus and clypeal pleurite, ventral **C** anterior part of cephalic pleurite, ventral **D** maxillae complex, ventral. Scale bars: 500 µm (**A, B**); 100 µm (**C**); 250 µm (**D**).

**Figure 9.**
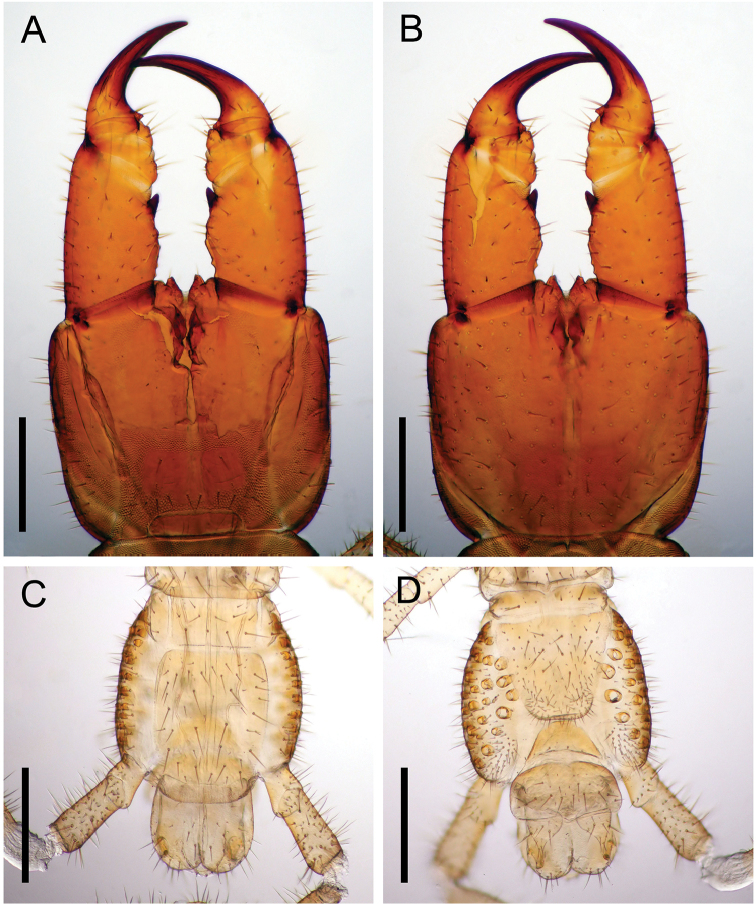
*Arrupakiyoshiensis* sp. n., **A, B** paratype (TS-20180418-01) **C, D** holotype (TS-20180330-01) **A** forcipular segment, dorsal **B** forcipular segment, ventral **C** last leg-bearing and terminal segments, dorsal **D** last leg-bearing and postpedal segments, ventral. Scale bars: 500 µm.

###### Distribution.

Known from only the type locality.

###### Type locality.

Kagekiyo-ana, Mitou Town (Mitou-cho), Mine City (Mine-shi), Yamaguchi Prefecture, Japan (34°17.50'N, 131°20.00'E).

###### Remarks.

**Figure 10.**
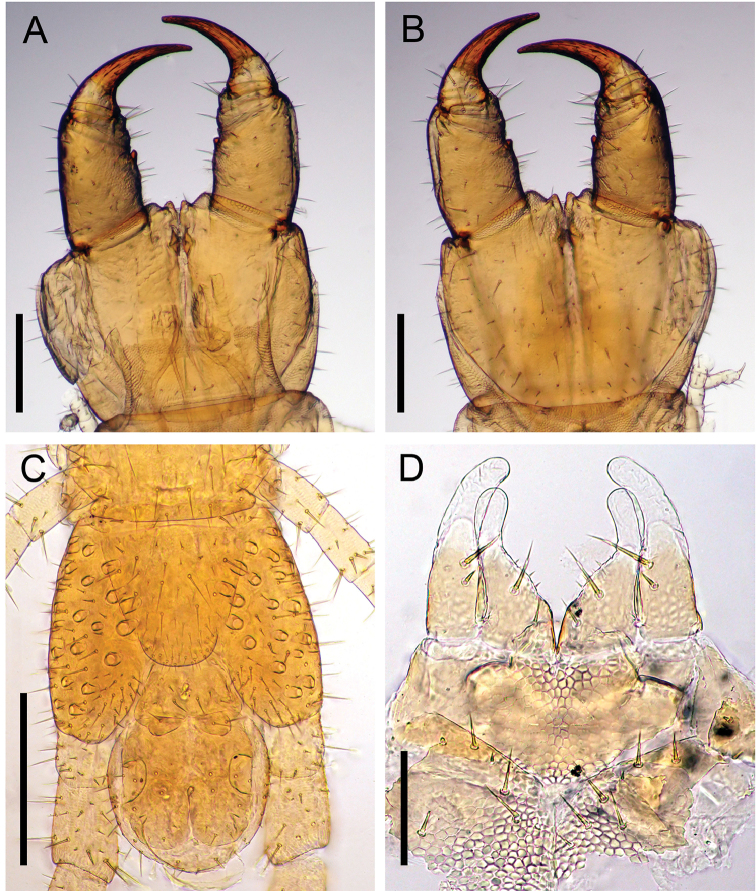
*Arrupishiianus* Uliana, Bonato & Minelli, 2007 (TS-20090729-01) **A** forcipular segment, dorsal **B** forcipular segment, ventral **C** last leg-bearing and postpedal segments, ventral **D** maxillae complex, ventral (telopodites of second maxilla broken). Scale bars: 300 µm (**A–C**); 100 µm (**D**).

*Arrupakiyoshiensis* sp. n. is morphologically similar to several other congeners, especially *A.holstii* (Pocock, 1895) and *A.ishiianus* Uliana, Bonato & Minelli, 2007 (Fig. 10), but can be easily distinguished from them by a combination of the characteristics shown in Table 7.

**Table 7.** Morphological comparison between *A.akiyoshiensis* sp. n. and other similar congeners based on Uliana et al. (2007).

Characteristics	*A.akiyoshiensis* sp. n.	* A. holstii *	* A. ishiianus *	* A. obtusus *	* A. kyushuensis *	* A. longicalix *
Body length	approx. 3.5 cm	approx. 2 cm	4–5 cm	approx. 2 cm	1.5–3 cm	approx. 2 cm
Shape of the distal lobe of medial projection	clavate at the top	clavate at the top	slightly clavate	–*	slightly clavate	very elongate
distal tooth of forcipular article I	well developed, pointed	sharp and short	well developed, rounded	well developed	large and subtriangular	pointed, medium sized
basal tooth of forcipular tarsungulum	triangular	sharp	rounded or slightly pointed	shallow and rounded	well developed	very shallow and obtuse
Sternite of ultimate leg-bearing segment	as long as wide	as long as wide	wider than long	wider than long	as long as wide	wider than long
Number of coxal pore	31–35	around 12	around 35	around 40	–*	around 15

* no data in Uliana et al. (2007).

## Discussion

The two adult female specimens examined were morphologically almost identical (except for the body size), and were therefore concluded to be conspecific. Male characteristics are unknown at present.

*Arrupakiyoshiensis* sp. n. exhibits unique characteristics which are not observed in other valid named congeners, i.e., the entire areolation of the crypeal pleurite, elongation of distal part of female gonopod, and tiny tubercle on forcipular article II. It is most similar to *A.holstii* (Pocock, 1895) and *A.ishiianus* Uliana, Bonato & Minelli, 2007 (Fig. 10) known from Japan, but can be easily distinguished from them by clearly developed, outwardly pointed tooth of the forcipular segment I (sharp and short tooth in *A.holstii*; well developed, rounded tooth in *A.ishiianus*), and triangular basal tooth of forcipular tarsungulum (sharp basal tooth in *A.holstii*; rounded or slightly pointed basal tooth in *A.ishiianus*). It is also easily distinguished from other similar congeners (Uliana et al. 2007) by a combination of the characteristics shown in Table 7. In addition, the 18S rRNA gene sequences of *A.akiyoshiensis* sp. n. and *A.ishiianus* differed by four bp out of 1821 bp (sequence of three individuals of *A.akiyoshiensis* sp. n. are all identical). Therefore, the undetermined species is herein concluded to be new species, named *A.akiyoshiensis*.

*Arrupakiyoshiensis* sp. n. and *A.holstii* can be found in the same area, Akiyoshi-dai. However, it is unclear whether the both species occur in the same cave or not, because the cave where *A.holstii* was found is not clearly mentioned (Kuramoto 1980). At least, *A.holstii* was not collected in our survey of Kagekiyo-ana, where *A.akiyoshiensis* sp. n. was collected. Many endemic species with small geographic ranges may occur in isolated caves (Barr Jr and Holsinger 1985). For further understanding of endemism of Akiyoshi-dai, a thorough inventory of cave invertebrates is needed.

*Arrupakiyoshiensis* sp. n. has no troglomorphic traits such as exceptionally long antennae, legs, and claws (Stoev et al. 2015). In the collection site (shown in Fig. 1), four troglobionts species *Nesticusakiyoshiensisakiyoshiensis* (Uyemura, 1941) (Araneae); *Coecobryaakiyoshiana* (Yosii, 1956) (Collembola); *Trechiamaplutokanekiyo* Ueno, 1958 (Coleoptera); an undescribed species of Campodeidae (Diplura), and one troglophiles species: *Epanerchodusetoietoi* Miyosi, 1955 (Polydesmida) can be found. *Thereuopodaclunifera* (Wood, 1862) (Scutigeromorpha) and species of Rhaphidophorid (Orthoptera), both of which have strongly indicated epigeal ecology, cannot be found (T Murakami pers. obs.). Considering the above facts, the habitat of *A.akiyoshiensis* sp. n. seems to be confined to cave environments. Further surveys are needed to consider adaptations of its ecology for cave environment.

## Supplementary Material

XML Treatment for Arrup akiyoshiensis
